# BS GSA AND AGE JOINT SYMPOSIUM

**DOI:** 10.1093/geroni/igad104.1937

**Published:** 2023-12-21

**Authors:** Blanka Rogina, Rozalyn Anderson

## Abstract

Since 2020, BS GSA and American Aging Association (AGE) recognized the opportunity to present a joint session regularly at each organization’s national meeting. Given our unique positions in the field of aging research, we recognize the opportunity to advance each other’s missions and strengthen the ecosystem to advance translation of aging research into practice and policy. This symposium has been a resounding success and an ideal forum for early-stage investigators who seek to develop a program at the interface of basic and clinical research.

